# Insight into the Current Genetic Diversity and Population Structure of Domestic Reindeer (*Rangifer tarandus*) in Russia

**DOI:** 10.3390/ani10081309

**Published:** 2020-07-30

**Authors:** Veronika Kharzinova, Arsen Dotsev, Anastasiya Solovieva, Olga Sergeeva, Georgiy Bryzgalov, Henry Reyer, Klaus Wimmers, Gottfried Brem, Natalia Zinovieva

**Affiliations:** 1 L.K. Ernst Federal Science Center for Animal Husbandry, 60, Settl. Dubrovitsy, Podolsk Municipal District, 142132 Moscow Region, Russia; asnd@mail.ru (A.D.); anastastasiya93@mail.ru (A.S.); gottfried.brem@agrobiogen.de (G.B.); 2 Research Institute of Agriculture and Ecology of the Arctic—Branch of the FRC KSC SB RAS, 663302 Norilsk, Russia; ols-78@mail.ru; 3 Magadan Research Institute of Agricultural, 685000 Magadan, Russia; agrarian@maglan.ru; 4 Institute of Genome Biology, Leibniz Institute for Farm Animal Biology (FBN), Mecklenburg-Vorpommern, 18196 Dummerstorf, Germany; reyer@fbn-dummerstorf.de (H.R.); wimmers@fbn-dummerstorf.de (K.W.); 5 Institut für Tierzucht und Genetik, University of Veterinary Medicine (VMU), Veterinärplatz, A-1210 Vienna, Austria

**Keywords:** reindeer, breeds, genetic diversity, population structure, SNP

## Abstract

**Simple Summary:**

Reindeer herding is the most important agricultural sector of the Russian Far North, representing the local genetic resources that compose original genetic wealth for the indigenous Arctic ethnic groups, which has maintained their life in harsh conditions of the area for many years. Conservation about and further rational use of such resources are very difficult without taking into account genetic diversity. Here, for the first time, the current genetic composition of the four officially recognized reindeer breeds and their ecotypes inhabiting the area from the Kola Peninsula in the west to the Chukotka region in the east are described using a single-nucleotide polymorphism (SNP) array. Our findings reveal the genetic uniqueness of each breed, formed by the consequences of ecological processes, internal gene flow, breeding practices, and geographical features. The obtained results will assist the ongoing breeding policy to develop accurate programs to preserve genetic resources of this essential element of Russia’s Far North ecosystem.

**Abstract:**

To examine the genetic diversity and population structure of domestic reindeer, using the BovineHD BeadChip, we genotyped reindeer individuals belonging to the Nenets breed of the five main breeding regions, the Even breed of the Republic of Sakha, the Evenk breed of the Krasnoyarsk and Yakutia regions, and the Chukotka breed of the Chukotka region and its within-breed ecotype, namely, the Chukotka–Khargin, which is bred in Yakutia. The Chukotka reindeer was shown to have the lowest genetic diversity in terms of the allelic richness and heterozygosity indicators. The principal component analysis (PCA) results are consistent with the neighbor-net tree topology, dividing the reindeer into groups according to their habitat location and origin of the breed. Admixture analysis indicated a genetic structuring of two groups of Chukotka origin, the Even breed and most of the geographical groups of the Nenets breed, with the exception of the Murmansk reindeer, the gene pool of which was comprised of the Nenets and apparently the native Sami reindeer. The presence of a genetic component of the Nenets breed in some reindeer inhabiting the Krasnoyarsk region was detected. Our results provide a deeper insight into the current intra-breeding reindeer genetic diversity, which is an important requirement for future reindeer herding strategies and for animal adaptation to environmental changes.

## 1. Introduction

Reindeer (*Rangifer tarandus* L. 1758) is an ungulate that inhabits the whole circumpolar area of the northern hemisphere, and it is the last animal to be domesticated by humans [1,2]. There are currently about 2.5 million domesticated reindeer located across nine countries, among which the Russian Federation is the largest reindeer breeder. Reindeer herding is the only agricultural sector in the Arctic region, which is concentrated in 18 Russian Federation subjects of four federal districts [3]. The century-long process of purposeful implementation of the particular sets of practices in these districts, as well as scientifically based selection and the pure-line breeding within the Soviet Union era, are reflected in the creation of unique populations that differ from each other not only in exterior and constitution, but also in the nature of their phenotypic traits. Subsequently, in 1985, these differences were entrenched in order No. 212 of the Russian Ministry of Agriculture (formerly the USSR Ministry of Agriculture) by the official recognition of four reindeer breeds: Nenets, Even, Evenk, and Chukotka [4].

The Nenets breed (Figure S1) is the largest in Russia by number (1,300,800 heads) and pasture territory (110 million ha) [5]. These reindeer are of an average size with rather well-defined working capacities and high reproductive performance [6]. The Evenk breed (Figure S2) is considered to be the oldest, with the total stock amounting to no more than 20,000 animals [7,8]. These animals are known for their high load-carrying capacity and endurance, and are still extensively used for transport [9]. The reindeer of the Even breed (Figure S3) are well adapted to mountainous areas, occupying alpine pastures in summer and river valleys and depressions in winter [9]. The total stock of this breed amounts to approximately 154,000 individuals. A high meat production, as well as a high adaptation to the environmental conditions of the arctic and subarctic tundra and their well-developed herd instinct are inherent to the Chukotka reindeer breed (Figure S4), with the total stock of this breed amounting to 202,100 individuals [9,10].

Furthermore, due to differences in natural and economic conditions, specific feeding, and breeding systems, several within-breed ecotypes have been developed in different areas [11]. Yuzhakov et al. [12] highlighted several ecotypes within the Nenets breed, division into which is based on phenotypic traits (i.e., fawning term, constitutional peculiarities, body weight, and color). Four ecotypes of animals are distinguished in the Chukotka breed, as referred to by Baskin [13], three of which are bred in areas of the Kamchatka Peninsula and the Chukotka region, while the fourth one inhabits two encampments of the tundra zone of Yakutia and is called Khargin. However, these reindeer had already been differentiated by their conformation, feeding habits, and behavior from their Chukotka conspecifics.

Moreover, considering the country’s economy, regional reindeer herding is concentrated in the seven regional clusters, which cover approximately 90% of all of the Russian reindeer, including the Murmansk region (i.e., European Russia, tundra reindeer herding on the Kola Peninsula by Saami and Komi with mixed Saami–Nenets reindeer), the Nenets Autonomous district and the Arkhangelsk region (European Russia, mainly tundra reindeer herding by European Nenets with the Nenets reindeer), the Komi Republic (European Russia, reindeer herding at the forest–tundra margin and mountains by Komi and Nenets with the Nenets reindeer), Yamalo-Nenets Autonomous district (West Siberia, mainly tundra reindeer herding, but also taiga, forest–tundra margin, and mountain types by the Nenets, Khanty, and Komi with the Nenets reindeer), the Sakha Republic (Yakutia; East Siberia, tundra, taiga, and forest types, by the Evenk (taiga) the Even (taiga, mountain, and tundra), the Sakha and Dolgans (mainly tundra) with the Evenk, Even, and Khargin reindeer), the Chukotka region (Far East, tundra type, mainly by Chukchi with Chukotka reindeer), and the Koryak Autonomous district (Far East, forest–tundra margin by Koryaks with Chukotka and Even reindeer) [14].

Despite the widespread occurrence of the domestic reindeer in the regions of the Russian Far North, the population census size over the last few decades has decreased. The first accurate registration of the reindeer population was carried out in 1926–1927, with the number of heads being 2,202,700, which then declined with collectivization to 1.4 million, stabilized after World War II, and later climbed back to very high levels of more than 2.1 million during the three sovkhoz decades (1961–1993) [14,15]. The recent drastic decline, which led authors such as Baskin [16] to consider domestic reindeer herding as being on the edge of extinction, happened after the Soviet Union collapse, reaching numbers lower than those of the early 20th century. As of 1 January 2019, the domestic reindeer population size was confirmed to be 1,702,000 livestock units [3].

The negative effects of the domestic reindeer decline might have reflected on processes such as genetic drift, gene flow, and natural selection, which, in turn, affect the distribution of the genetic diversity and structuring across a species range [17]. According to Lewis et al. [18], the study of the genetic diversity found within domestic animals can result in the most complete representation of species domestication, as well as mammalian biology and evolution. Additionally, the insight into the genetic variability pattern of breeds, especially those uniquely adapted to harsh climate conditions and the open housing system, is a crucial requirement for their sustainable conservation and for future animal breeding strategies [19,20].

Recent improvements in the speed, cost, and accuracy of next-generation sequencing have contributed to an obtainment of new information about the whole genome of most domestic animals, and have led to the widespread use of single-nucleotide polymorphism (SNP) markers as a powerful tool for addressing a great number of genetic tasks [21,22]. The discovery and genotyping of hundreds of thousands of SNPs in a single assay are enabled by the use of SNP high-throughput arrays, which are available for the majority of the livestock species, thus targeting wide genetic variants that are spread along the entire genome [23,24]. In distinction from the other model species, a genomic era in the study of both the reindeer and the North American caribou has been established recently, and several versions of the *Rangifer tarandus* genome have occurred [25,26,27]. The first genome assembly was announced in 2017 by Li et al. [25] and the latest update was in 2020 by a group of Scandinavian scholars (*Rangifer tarandus*—assembly RanTarSib_v1_BIUU) [27]. Meanwhile, the assignment of reindeer genome scaffolds to their specific chromosomes, as well as the array-based methods in which panels of predetermined polymorphisms are hybridized onto chips by companies such as Affymetrix or Illumina, for the species are still inaccessible. The lack of SNP microarrays for non-model organisms, as put forward by More et al. [28], has led to testing commercially available SNP microarrays of closely related species to discover common SNPs.

Despite the abundance of scientific work on the genetic biodiversity of the Cervidae family members through commercial SNP genotyping arrays [4,29,30,31,32]—including our previous study, which attempted to determine the differences among the domestic reindeer groups within the Nenets Autonomous district, the Murmansk region, and Yakutia, as well as among wild tundra and taiga forms inhabiting Yakutia and western Taymyr—information on the genetic structure and diversity of all domestic reindeer breeds inhabiting the Russian Far North is still lacking.

The erosion of domestic animal diversity due to natural causes and creative human activity is of serious concern if current production levels are to be sustained and the changing demands of future markets are to be addressed [33]. Thus, the clarification of the genetic relationships between domestic reindeer breeds requires a new scientific approach to its study.

Herein, using genome-wide SNPs generated from the BovineHD BeadChip, we aimed to address the primary issue: What is the current genetic diversity and structure of the four officially recognized breeds and their ecotypes inhabiting the reindeer herding regions, from the Kola Peninsula in the west to the Chukotka region in the east. We also aimed to answer a secondary question: Are the breed ecotypes differentiated into groups according to their habitats? The obtained results will assist the ongoing breeding policy to develop accurate programs to preserve genetic resources of this essential element of Russia’s Far North ecosystem.

## 2. Materials and Methods

### 2.1. Ethics Statement

The principles of laboratory animal care were followed, and all procedures were conducted according to the ethical guidelines of the L.K. Ernst Federal Science Center for Animal Husbandry. The protocol was approved by the Commission on the Ethics of Animal Experiments of the L.K. Ernst Federal Science Center for Animal Husbandry (Protocol Number: 2020/2). Biomaterials from the genetic resource collection of the L.K. Ernst Federal Science Center for Animal Husbandry, supported by the Ministry of Science and Higher Education of the Russian Federation, were used in this study. The tissue samples of domestic reindeer were collected by trained personnel under strict veterinary rules. The muscle tissue samples of wild reindeer were collected during scientific expeditions after obtaining collection permits granted by the Department of Hunting of the Republic of Sakha and the Taymyr Dolgano-Nenetsky District, in compliance with the Russian Federation Law No. 209-FZ of 24 July 2009.

### 2.2. Sample Collection and DNA Extraction

**Figure 1:**
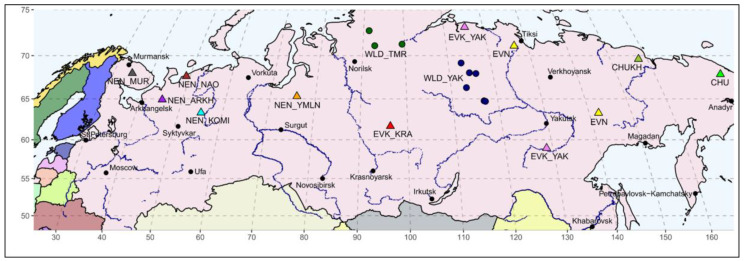
A geographical distribution of the sampled domestic and wild reindeer individuals on a map of Russia. CHU, Chukotka; CHUKH, Chukotka–Khargin; EVN, Even; EVK_YAK, Evenk of the Yakutia region; EVK_KRA, Evenk of the Krasnoyarsk region; NEN_YMLN, Nenets of the Yamalo-Nenets Autonomous district; NEN_NAO, Nenets of the Nenets Autonomous district; NEN_KOMI, Nenets of the Komi Republic; NEN_ARKH, Nenets of the Arkhangelsk region; NEN_MUR, Nenets of the Murmansk region; WLD_YAK, wild reindeer of Northern Yakutia; WLD_TMR, wild reindeer of Western Taymyr.

In total, 113 reindeer individuals representing four breeds were chosen for the current study, including the Nenets breed from the Nenets (NEN_NAO, *n* = 31) and Yamalo-Nenets (NEN_YMLN, *n* = 13) Autonomous districts, the Komi Republic (NEN_KOMI, *n* = 13), and the Arkhangelsk (NEN_ARKH, *n* = 10) regions; the Even breed of the Republic of Sakha (Yakutia) (EVN, *n* = 6); the Evenk breed of the Krasnoyarsk region (EVK_KRA, *n* = 19); and the Chukotka breed of the Chukotka Autonomous district (CHU, *n* = 12) and its within-breed ecotype—the Chukotka–Khargin (CHUKH, *n* = 9), which is bred in the Republic of Sakha (Yakutia). The biomaterial was picked during the corral work on the herd throughout 2018–2019. Additionally, our data set was combined with SNP genotypes of reindeer that were obtained during our previous study [4], namely, the Nenets breed from the Nenets Autonomous district (NEN_NAO, *n* = 33) and the Murmansk region (NEN_MUR, *n* = 20) and the Evenk (EVK_YAK *n* = 12), the Even (EVN, *n* = 7), and the Chukotka–Khargin breeds (CHUKH, *n* = 2) from the Republic of Sakha (Yakutia). All studied reindeer groups are displayed in Figure 1.

In addition, considering the peculiarity of reindeer species, namely, the coexistence of domestic and wild forms, and to find out a possible genetic contribution toward the domestic reindeer population structure, the wild individuals were included in the analyses as an outgroup. The muscle tissue samples were taken from the wild reindeer inhabiting the Western Taymyr (WLD_TMR, *n* = 21) and the Northern Yakutia (WLD_YAK, *n* = 11). The coordinate range of the covered area in Taymyr varied from 70 to 73° N and from 91 to 99° E; in Yakutia, it ranged from 64 to 69° N and from 113 to 117° E. Collections of the wild samples were taken from 2017 to 2019 during scientific expeditions.

The geographic map (with longitude and latitude coordinates for each sampling site) was plotted using the R packages maps and mapdata [34].

Genomic DNA was extracted with Nexttec columns (Nexttec Biotechnology GmbH, Germany) following the manufacturer’s instructions. The DNA samples were quantified using a method of visualization in bands by 1% agarose gel electrophoresis. The concentration of the dsDNA was measured on a Qubit 3.0 fluorimeter (Thermo Fisher Scientific (formerly Life Technologies), Wilmington, DE, USA). DNA purity was determined by evaluating the absorption ratio of A260/A280 on a NanoDrop2000 spectrophotometer (Thermo Fisher Scientific, Wilmington, DE, USA).

### 2.3. Genotyping and SNP Quality Control (Genotype and SNP Calling)

DNA samples were genotyped using the Illumina BovineHD Genotyping BeadChip. The subsequent analysis first involved a filtering step in which the GenCall (GC) and GenTrain (GT) scores were used. A cut-off of 0.5 for both the GC and GT scores was applied to determine the valid genotypes for each SNP [35]. SNPs located on the sex chromosomes of the UMD 3.1 assembly [36], as well as SNPs with unknown map positions, were discarded. Genotype quality control (QC) procedures were performed using PLINK v1.90 [37]. SNPs with a genotyping rate of >90% across the data set were identified using the command --geno 0.1. SNPs with a minor allele frequency less than 5% (--maf 0.05) and SNPs with a value of linkage disequilibrium (LD) between a pair of single-nucleotide polymorphisms equal to r^2^ > 0.05 (we used a sliding window of 50 SNPs, sliding along in 5 SNP increments) were excluded from further analyses. The final data set was accomplished by removing the individuals with more than 10% missing genotypes (--mind 0.1). As a result, 7968 SNPs passed quality control filtering, while 641,698 variants were removed due to missing genotype data (--geno 0.1) and 85,627 variants were removed due to a minor allele threshold (--maf 0.01). After LD pruning, 665 SNPs were removed, and we obtained a final set of SNPs containing 7303 markers.

### 2.4. Genetic Diversity

To measure the level of genetic diversity in each reindeer breed, the observed heterozygosity (*H*_O_), the unbiased expected heterozygosity (_U_*H*_E_) [38], the inbreeding coefficient (*F*_IS_) based on the unbiased expected heterozygosity, and the rarefied allelic richness (*A*_R_) were computed with the R package “diveRsity” [39].

### 2.5. Population Structure Analysis

To discover the genetic structure of the studied reindeer breeds, we implemented a principal component analysis (PCA) in PLINK v1.90, visualized with the R package “ggplot2” [40].

ADMIXTURE v1.23 software [41] was used to reveal admixture patterns among the reindeer groups. The optimal value of K was determined based on the lowest cross-validation error compared to those of the other K values. We evaluated K values ranging from 1 to 10. The graphical representation of the admixture patterns was depicted using the R package “pophelper” [42]. 

### 2.6. Population Differentiation and TreeMix Analysis

To describe the differentiation of the reindeer breads, pairwise *F*_ST_ measures were calculated and the computing process was completed in the R package diveRsity. A pairwise distance matrix of *F*_ST_ measures was applied for construction of a consensus neighbor-net tree through the SplitsTree 4.14.5 software [43].

TreeMix 1.12 package software [44] was used to measure both population splits and the extent of gene exchange between reindeer breeds. As wild reindeer were included in the data set, both populations (WLD_TMR and WLD_YAK) were assumed as a rooting outgroup. We sequentially tested up to five migration edges and ran 100 independent interactions for each edge. Standard errors (-se) and *p*-values were calculated with jackknife blocks of 100 SNPs (-k 100). We found four migration edges as the most likely number of migration events based on the log-likelihood of the event (*p* < 0.05).

All input files were created in R version 3.3.2 [45].

## 3. Results

### 3.1. Genetic Diversity

Genetic diversity is shaped by past population processes and affects the sustainability of species and populations in the future [46]. The mean number of alleles (*A*_R_), as well as the observed (*H*_O_) and unbiased expected _U_*H*_E_ heterozygosity, which are commonly measured parameters to check within-breed diversity [47], were calculated for the studied reindeer set and are presented in Table 1.

Allelic diversity, as measured by allelic richness, ranged from 1.573 to 1.666, with CHU showing the lowest value and EVK_YAK the highest. Regarding the heterozygosity indices, six groups were characterized by a value of observed heterozygosity above 0.180, with the exception of CHU, CHUKH, NEN_YMLN, and NEN_ARKH, in which the value of the indicator was below 0.179. For expected heterozygosity (unbiased to sample size), seven groups had a high comparable value, ranging from 0.183 for NEN_YMLN to 0.192 for EVK_YAK. A lower level of the indicator was detected for CHU (0.176), CHUKH (0.179), and NEN_ARKH (0.179). A slightly positive *F*_IS_ value was observed in all groups, ranging from 0.012 for CHU and EVK_YAK to 0.027 for EVN.

### 3.2. Population Differentiation and TreeMix Analysis

The distance analysis based on the differentiation coefficients (*F*_ST_) showed low variable *F*_ST_ values, ranging from 0.003 to 0.096 (Table 2).

The lowest *F*_ST_ values were observed for geographically close reindeer groups, namely, for the Nenets breed, which are kept on farms in the Komi Republic and the Nenets and Yamalo-Nenets regions: NEN_KOMI/NEN_YMLN (0.003), NEN_NAO/NEN_YMLN (0.004), and NEN_KOMI/NEN_NAO (0.005). The next lowest level of differentiation was detected between the Chukotka breed and its ecotype from Yakutia region, CHU/CHUKH (0.01), and between two breeds inhabiting the Yakutia region: EVK_YAK/EVN (0.017). The highest genetic differentiation of 0.096 was observed between the Archangelsk population of the Nenets breed and a group from the Chukotka region (NEN_ARKH/CHU). Notably, a practically equal low connectivity (varied from 0.08 to 0.096) was revealed between the four groups of the Nenets breed and the reindeer of Chukotka origin raised in both the Chukotka and Yakutia regions.

**Figure 2:**
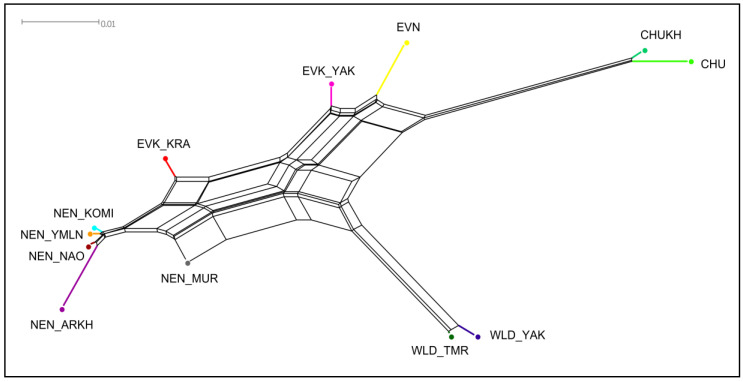
Neighbor-net tree of the Russian reindeer breeds based on pairwise *F*_ST_ values. CHU, Chukotka; CHUKH, Chukotka–Khargin; EVN, Even; EVK_YAK, Evenk of the Yakutia region; EVK_KRA, Evenk of the Krasnoyarsk region; NEN_YMLN, Nenets of the Yamalo-Nenets Autonomous district; NEN_NAO, Nenets of the Nenets Autonomous district; NEN_KOMI, Nenets of the Komi Republic; NEN_ARKH, Nenets of the Arkhangelsk region; NEN_MUR, Nenets of the Murmansk region; WLD_YAK, wild reindeer of Northern Yakutia; WLD_TMR, wild reindeer of Western Taymyr.

Further, based on the *F*_ST_ values, more precise genetic pathways of the connection between the studied reindeer breeds were reflected in the neighbor-net tree (Figure 2).

Two conventional clusters were noted in the network. The core of the first cluster was presented by the populations of the Nenets breed (except for NEN_MUR) with the joining of the EVK_KRA and NEN_MUR branches. Additionally, the NEN_ARKH reindeer were fractionally positioned outside of the Nenets clade. Regarding the second cluster, three subclusters were formed, composing a branch of two breeds raised in the Yakutia region (EVK_YAK and EVN), a group of the wild populations (WLD_TMR and WLD_YAK), and the reindeer of Chukotka origin (CHUKH and CHU), which occupied the opposite location on the tree.

**Figure 3:**
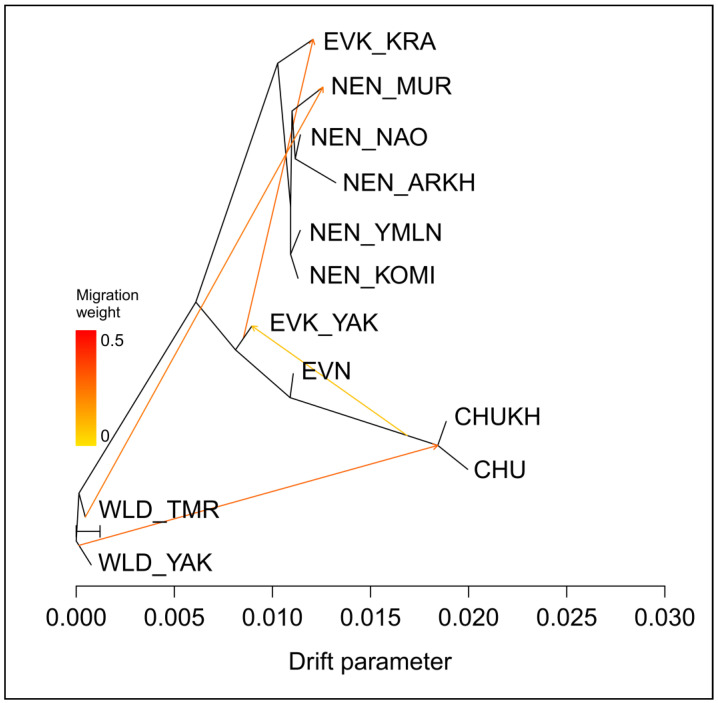
Maximum likelihood tree produced by TreeMix with four migration edges. ^1^ CHU, Chukotka; CHUKH, Chukotka–Khargin; EVN, Even; EVK_YAK, Evenk of the Yakutia region; EVK_KRA, Evenk of the Krasnoyarsk region; NEN_YMLN, Nenets of the Yamalo-Nenets Autonomous district; NEN_NAO, Nenets of the Nenets Autonomous district; NEN_KOMI, Nenets of the Komi Republic; NEN_ARKH, Nenets of the Arkhangelsk region; NEN_MUR, Nenets of the Murmansk region; WLD_YAK, wild reindeer of Northern Yakutia; WLD_TMR, wild reindeer of Western Taymyr.

We estimated the most likely evolutionary history of each group of the domestic reindeer populations by calculating the levels of genetic drift at a set of 7303 SNPs. To this end, the TreeMix program was used, and its topology with four migration edges is shown in Figure 3.

The most likely migration edge indicated the existence of a gene exchange event from WLD_YAK to the group of Chukotka origin. The second and the third migration edges with a milder migration event denoted a gene flow from WLD_TMR to NEN_MUR and from EVK_YAK to EVK_KRA. The fourth migration edge with the smallest weight indicated a gene flow between the groups of Chukotka origin to the Evenk breed of Yakutia.

### 3.3. Population Structure Analysis

**Figure 4:**
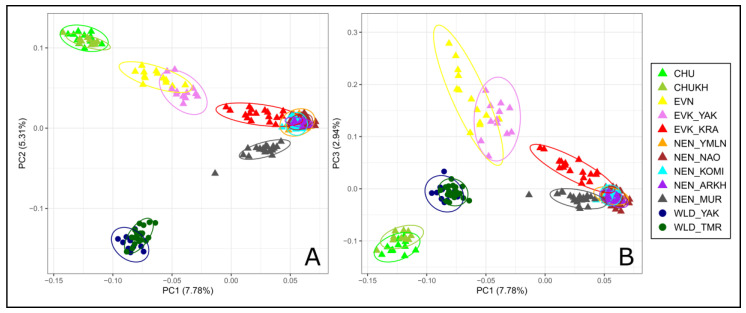
Principal component analysis (PCA) analysis of the Russian reindeer breeds: (**A**) the first two components (PC1 and PC2), which, together, explain 13% of the genetic variability and (**B**) the first and third components (PC1 and PC3), which, together, explain 10.7% of the genetic variability. ^1^ CHU, Chukotka; CHUKH, Chukotka–Khargin; EVN, Even; EVK_YAK, Evenk of the Yakutia region; EVK_KRA, Evenk of the Krasnoyarsk region; NEN_YMLN, Nenets of the Yamalo-Nenets Autonomous district; NEN_NAO, Nenets of the Nenets Autonomous district; NEN_KOMI, Nenets of the Komi Republic; NEN_ARKH, Nenets of the Arkhangelsk region; NEN_MUR, Nenets of the Murmansk region; WLD_YAK, wild reindeer of Northern Yakutia; WLD_TMR, wild reindeer of Western Taymyr.

PCA and the model-based approach implemented in Admixture software were used to infer the population structure of the studied reindeer groups. The PCA results (Figure 4) indicated a genetic differentiation between the breeds and united the reindeer into clusters that were consistent with the neighbor-net tree.

The reindeer from the Murmansk region formed an independent cluster, while the remaining individuals of the Nenets breed displayed a convergent pattern of their genetic composition, forming a slightly overlapping cluster with EVK_KRA. Additionally, the analysis divided the remaining groups into three clusters: the Even and the Evenk breeds inhabiting the Yakutia region formed one cluster, while the wild populations formed another cluster, and the Chukotka and the Chukotka–Khargin groups formed the last cluster. The contributions of the total genetic variability, accounted by the first, second, and third components, were 7.78%, 5.31%, and 2.94%, respectively. All individuals of the Nenets population, as well as the Evenk reindeer from the Krasnoyarsk region, were distributed along the first principal component, which distinguished them from the remaining reindeer. The second principal component highlighted the divergence of the domestic and wild populations with the assignment of the Murmansk individuals to the wild relatives. Further, the third principal component split the reindeer of Chukotka origin and the Nenets breed’s representatives from the Even and the Evenk breeds. Meanwhile, the reindeer of the wild populations were placed on the C3 axis.

**Figure 5:**
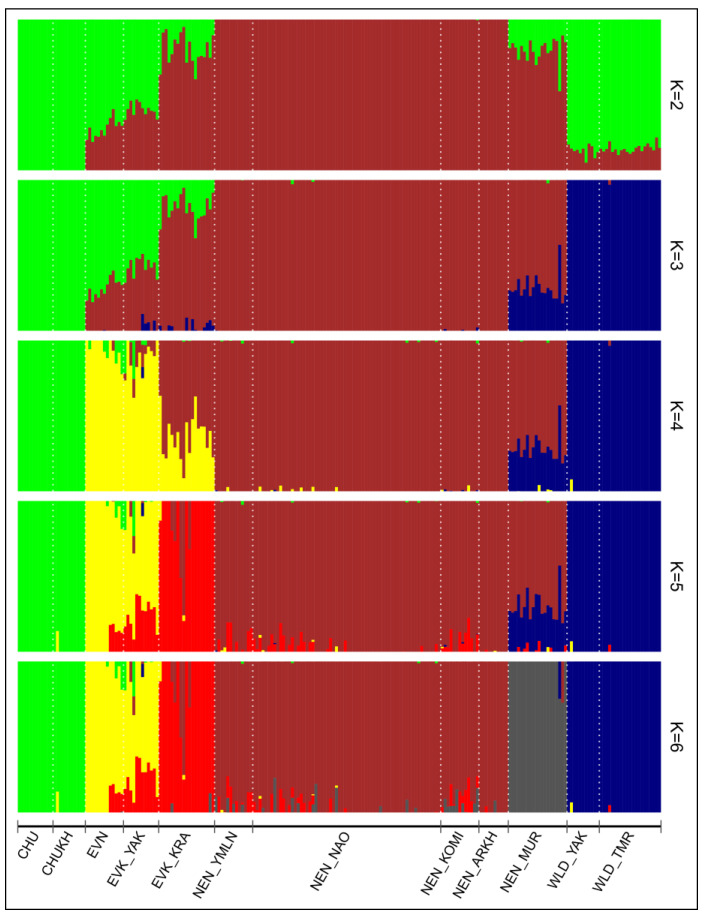
Admixture analysis of the Russian reindeer breeds. CHU, Chukotka; CHUKH, Chukotka–Khargin; EVN, Even; EVK_YAK, Evenk of the Yakutia region; EVK_KRA, Evenk of the Krasnoyarsk region; NEN_YMLN, Nenets of the Yamalo-Nenets Autonomous district; NEN_NAO, Nenets of the Nenets Autonomous district; NEN_KOMI, Nenets of the Komi Republic; NEN_ARKH, Nenets of the Arkhangelsk region; NEN_MUR, Nenets of the Murmansk region; WLD_YAK, wild reindeer of Northern Yakutia; WLD_TMR, wild reindeer of Western Taymyr.

According to a cross-validation procedure, the best predictive accuracy of the model was detected for K = 3 (CV, 0.355) and for K = 2 (CV, 0.358; Figure S5). However, significant results of reindeer clustering, from our point of view, were additionally noticed at K = 4, K = 5, and K = 6 (Figure 5), which are presented as well.

At K = 2, two main genetic pools were revealed; the first one was formed by the three breeds inhabiting the Yakutia region, the Chukotka breed, and the wild populations, whereas the second pool comprised the Nenets regional populations and the Evenk breed from the Krasnoyarsk region. It is worth noting that EVN and EVK_YAK had a high proportion of admixture in their individuals from the second genetic pool, the independent ancestry of which became evident at K = 4. Interestingly, the Evenk reindeer from Yakutia did not form an independent cluster and, moreover, demonstrated a slight admixed ancestry with the same breed raised in the neighboring Krasnoyarsk region at K = 5. The admixture origin of the Krasnoyarsk reindeer, in return, was maintained until K = 5, at which they showed an independent ancestry, albeit with the genetic component of the remainder of the Nenets breed in some individuals. Meanwhile, at K = 3, a proportion of admixture of the wild gene component was detected in the individuals from the Murmansk region, which constituted a complete separate group at K = 6. The Chukotka reindeer of the two breeding regions maintained a common genetic background from K = 2 to higher values.

## 4. Discussion

In comparison to other livestock, reindeer herding is still the most important agricultural sector of the Russian Far North. Despite gradually replacing local breeds with high-yield culture ones, in the vast majority of geographical places, reindeer is one of the few species of domesticated animals that has acquired adaptive traits to survive in the extreme natural conditions of the Arctic. The conservation and further sustainable use of such resources are very difficult without considering genetic diversity. In this regard, the main aim driving our study was the examination of the current genetic composition of four officially recognized breeds, as well as their ecotypes, inhabiting the four federal districts of the Russian Far North. Additionally, we questioned the genetic divergence of the breeds’ ecotypes into groups according to their habitats due to breeding practices and geographical features.

To address the above challenges, in the current study, the reindeer individuals were genotyped with the Illumina BovineHD Genotyping BeadChip, and the population genetic indices were calculated based on 7303 SNPs. The first utilization of the BovineHD BeadChip in *Rangifer* was reported in 2018 [4]. The study was designed to investigate the genetic differences of the regional domestic and wild populations inhabiting Northeast Russia, since this is an important aspect in the conservation of both groups. The final data set comprised 8357 SNPs for domestic and 8145 SNPs for wild individuals. Kasarda et al. [30], using 5.3% polymorphic SNPs from the BovineSNP50 BeadChip, successfully evaluated genetic diversity and genetic relatedness among three groups of cervids. Genetic differences between farmed and free-range Red deer originating from Slovakia were observed based on 1530 polymorphic markers [48]. Despite some of the cautions associated with the use of commercially developed arrays for genetically related species [29], as well as with the use of a species-specific DNA Chip to provide more accurate characteristics of the animal genome, even a small proportion of polymorphic cross-amplifying SNPs allows us to infer both the population structure and the variability across and within individuals, for which commercial chips have not yet been developed.

Knowledge of the level and distribution of genetic diversity is the basis for a species’ survival, development, and evolution, and for improvement programs in animal production [49]. One of the measures of genetic diversity, which assists in understanding the population’s long-term potential for adaptability and persistence, is allelic richness [50].

The results of the present study showed that the Evenk and Even breeds raised on the farms of the Yakutia region exceeded their domestic relatives in this indicator (Table 1). According to Bashalkhanov et al. [51], the observed high amount of the allelic richness, which increases almost exclusively by rare alleles, indicates a higher potential for the populations to adapt to changing environmental conditions. Additionally, both groups were characterized by a high level of genetic variability in terms of the observed and expected heterozygosity (Table 1). It is possible that this could be the result of one or more past evolutionary events, caused by the pasturage and migration ways of herds from different households of the region, crossed in either way, and, thus, gene exchange could not be excluded. Interestingly, the close kinship of the Even and Evenk reindeer of the Yakutia region was depicted in the neighbor-net tree and the PCA plot (Figure 2 and Figure 4). There is evidence that until the 19th century, the Yakuts usually kept their few domestic reindeer in herds of the neighboring ethnic group [52]. Such cooperation was caused by a characteristic feature of the economic organization of this ethnic group [53]. On the other hand, an independent ancestry of the Even breed and clear admixture proportions between the EVK_YAK and EVK_KRA gene pools were found by the results of admixture (Figure 5). Moreover, a gene flow from EVK_YAK to EVK_KRA was illustrated by the third migration edge of TreeMix (Figure 3). The observed patterns may be associated with the history of the breed in terms of their occurrence in the region. A common feature of EVN and EVK_YAK, in addition to being bred on the same territory, is their belonging to the Tungus–Yakut reindeer husbandry type. In the past, as reported by Jochelson [54], the natural diversity and the small population of the Yakutia region caused the appearance of the Tungus tribes from the East Sayan region. Their reindeer had morphological features that are now characteristic of the Evenk breed (i.e., height of leg and coat color). Later, more numerous and warlike than the Tungus, herder ancestors of the Yakuts appeared, which caused the displacement of the Tungus to the periphery of these lands [55,56]. The Yakuts acquired their reindeer from the Tungus, but in spite of this, they applied to their herds the method of conscious selection with which they were familiar as horse and cattle breeders. These reindeer differ from those of the Tungus in every way: they are larger, stronger, and tamer. The results of this selection process are also apparent in the color of the hair, and there are many spotted animals, the skins of which are the most highly valued, and they are trained for riding and driving [54].

In this study, we found that the Even appeared to be the most genetically consolidated breed. The Evenk reindeer of the Yakutia region had admixed patterns with the EVK_KRA and the EVN genetic components, caused both by the common ancestry with the Evenk conspecifics of the Krasnoyarsk region and the gene flow between the Even and Evenk herds being raised in the same region.

One more group of reindeer among the studied domestic breeds, which also belongs to the same reindeer husbandry type, is the Evenk of the Krasnoyarsk region [15]. This group presented an average level of genetic variability (Table 1) and is characterized by a particular structure. The neighbor-net tree placed this group on an independent branch between the NEN and EVK_YAK breeds (Figure 2). Nevertheless, on the PCA plot, we observed a slightly overlapping cluster of this group with the populations of the Nenets breed (Figure 4). Likewise, the admixture analysis (Figure 5) depicted the presence of the genetic component of the Nenets breed in some Krasnoyarsk individuals. The observed integration could have been caused by the occurrence of Nenets reindeer transportation to the Krasnoyarsk region in order to renew the gene pool of the Evenk breed [57].

In our study, the largest sample set was presented by the populations of the Nenets breed, due to the fact that this is the largest breed by both numbers and pasture territory, and due to the several phenotypes within the breed [12]. The values of the genetic diversity parameters were distributed almost identically between groups, except for the reindeer from the Arkhangelsk region, which showed the lowest *A*_R_, *H*_O_, and _U_*H*_E_ (Table 1). Furthermore, we noted that the Arkhangelsk reindeer were found on an independent branch of the Nenets breed cluster according to the tree population based on pairwise *F*_ST_ values (Figure 2), while the common genetic background shared between the remaining Nenets reindeer was indicated by the PCA and admixture results (Figure 4 and Figure 5). Meanwhile, our particular attention was taken by the reindeer from the Murmansk region. This group showed a distinctive genetic structure from the other regional populations, with the presence of the Nenets and wild gene components, as suggested by the results of the neighbor-net and the maximum likelihood trees and the population structure analysis (Figure 2, Figure 3, Figure 4 and Figure 5). Additionally, this conclusion was evidenced by a significant negative f3 statistic, which showed that the Murmansk reindeer originated from two ancestral groups: the Nenets breed raised in the Nenets Autonomous district and the wild population [4]. These detected distinctions of this domestic group can be interpreted by the origin of reindeer herding in the region. The Saami on the Kola Peninsula are the westernmost people herding reindeer in Russia [14]. The ubiquitous presence of hedges and pens for a small number of animals, which are in private use, indicates that hunting for the wild reindeer, for a long time, was the basis of economic prosperity and did not require worrying about the development of their own herd [58]. As referred to by Kozlov et al. [59], in the Saami, it is difficult to date the occurrence of the first herds of tamed and riding-trained reindeer. However, there is evidence that already in the 13th century, this ethnic group used a small number of the reindeer for transport purposes, and in the second half of the 17th century, they already had large herds. A typical feature of the reindeer husbandry of the Kola Sami almost until the end of the 19th century was the so-called free grazing in which reindeer were set free on the pastures for the whole summer and were collected only in autumn [14,58]. In 1886, families of the Komi-Izhemtsy for searching the reindeer pastures moved to the Kola Peninsula from the Arkhangelsk province with their own reindeer (9000 units), the arrival of which initiated the mixing of Saami and Nenets reindeer [60,61]. Since then, the purebred Saami reindeer group, which some authors call the Murmansk breed, started to be replaced by the Nenets breed [62].

The results of the current study found a genetic homogeny of most geographical groups of reindeer related to the Nenets breed, and thus, the existence of several within-breed ecotypes has not yet been revealed. An exception was detected for Murmansk reindeer, which belong to the Nenets breed, while their gene pool comprises the Nenets and apparently the native Sami reindeer. Moreover, a predominance of the Kola Peninsula reindeer in height at the withers and in live weight of calves at birth over their Nenets relatives was revealed [5]. The genetic structural similarity of the geographical populations of the Nenets breed was also confirmed by the microsatellite analysis [63] and the allelic variants of the transferrin locus [5].

Meanwhile, the reindeer of the Chukotka breed and its Yakut within-breed ecotype, which is called Khargin, had the lowest values for each genetic diversity parameter (Table 1). We noted that these two groups belong to the same cluster from K = 2 to a higher value at K = 6 (Figure 5), forming the overlapped clusters on the PCA plot (Figure 4) and placed in one branch on the neighbor-net network (Figure 2). Of note is the fact that the results of TreeMix (Figure 3) revealed the first migration edge from the wild reindeer to both groups of the Chukotka breed, thereby additionally confirming their common genetic origin.

The most likely reason to explain this discovered pattern of similar genetic structure of the groups is the difference of domestication processes of the Chukotka breed, with its within-breed ecotype, from the remaining breeds. Reindeer husbandry of the Chukchi originated later than among the Sami and Nenets people, as evidenced by the lack of tame reindeer. It is generally admitted that reindeer husbandry entered Chukotka only a few centuries ago and spread in the local tundra during the 17th–18th centuries [64]. Krushanov [65] reported that statistical information on the reindeer numbers in Chukotka at that time was still absent in comparison to the other domestic breeds. The presence of the majority of the Chukchi sledding and a small number of reproducing reindeer had only indirect evidence. Since the beginning of the 18th century, the Chukchi acquired reindeer by capturing them from the Koryak. Thus, Chukchi reindeer husbandry at the end of the 17th and in the first half of the 18th century was still far from becoming an independent branch of production, and it was organically merged with hunting. By the 1880s, the Chukchi raids on the Koryak ceased. Since then, reindeer husbandry in the region has been developing only due to natural growth. Furthermore, as reported by Baskin [13], the Chukchi moved to Yakutia with their reindeer, which were different in conformation, feeding habits, and behavior from that of their ancestors. However, despite this, our findings revealed that a current population of both the Chukotka and the Khargin reindeer are characterized by a mixed gene pool inherited from a common ancestor.

Evaluating the inbreeding and genetic diversity within a breed is a requisite for sustainable improvement in the long term [66]. In our study, we noted that the observed heterozygosity for all groups was practically close to the expected heterozygosity (Table 1), which denotes that the studied breeds were found to be under the Hardy–Weinberg (HW) equilibrium [67]. This assumption was additionally evidenced by a barely positive average inbreeding index, and the values of the confidence interval were almost tending toward zero, indicating the state of the genetic equilibrium in the reindeer groups. Of note is the fact that, in most of the domestic breeds, due to the intensive selection in economically oriented breeding schemes, increases in the rates of inbreeding were observed [66,68,69]. A discovered observation indirectly pointed out the differences between reindeer breeds and the remaining farm animals. It should be noted that in comparison to other livestock, reindeer herding is mainly carried out by traditional methods using visual estimates of morphological features. Besides, returning to history, these features of reindeer herding that differed from the strict definition of breeding, namely, artificial, caused scientific debates regarding a recognition of the breeds [70]. In the late 1970s, scientists such as Zabrodin et al. [9] concluded that the morphological differences between various domestic reindeer populations in Northern Russia were not sufficiently significant to constitute distinct breeds. On the other hand, according to Krupnik [71], in the 1700s reindeer herders were already able to manipulate the age–sex structure of the herds and the purposeful castration of bulls (for transport needs), which can be seen as forms of selection. Later, evidence of particular traits and behaviors of different breeds and their adaptability to their respective environments were put forward by Geist [72] and Yuzhakov et al. [73]. Stammler [14], when analyzing relationships between reindeer nomads and their social, political, and natural environments, outlined detailed differences between domestic breeds in terms of their main characteristics, such as migration inclination, foraging behavior, and suitability for meat and transport use.

In the present study, distinctions between the reindeer breeds were found not only in terms of allele and genetic diversity (Table 1), but in their genetic composition as well (Figure 2, Figure 3, Figure 4 and Figure 5). Our conclusions were found to be in accordance with the previous results, which revealed the genetic differences of some reindeer breeds based on studying transferrin locus polymorphisms [62], microsatellite variability [63,74,75], and SNPs [4,30,32].

## 5. Conclusions

In our study, using the BovineHD BeadChip, we provided current insight into the genetic variability pattern of four officially recognized breeds, as well as their ecotypes, inhabiting the four federal districts of the Russian Far North. Among all of the studied domestic groups, the Evenk and Even breeds raised on the farms of the Yakutia region were the most genetically variable, while the Chukotka breed and its Yakut within-breed ecotype, Khargin, had the lowest values for each genetic diversity parameter. For this breed, the turnover and conservation of genetic diversity can be advised. Regarding the genetic structure, we found that the reindeer breeds, in spite of their geographical habitats, maintained their specific genetic components. Additionally, based on our findings, we assumed that the genetic composition of the studied domestic reindeer groups is related to the features of their formation in the context of the ethnic history of the small peoples of the Far North.

Considering the assumption that the future genetic progress of the breeds depends mainly on the availability of sufficient genetic variation, we believed that the obtained results in this study will assist both the ongoing breeding policy and the development of a strategy for the conservation of this essential element of the life sustenance and culture of many indigenous and small peoples.

## Figures and Tables

**Table 1:** Measures of genetic diversity within domestic reindeer groups.

Breeds/ Population ^1^	n ^2^	Source of Sampling ^3^	A_R_^4^	H_O_^5^	_U_H_E_^6^	F_IS_^7^
CHU	12	The current study	1.573 ± 0.006	0.173 ± 0.002	0.176 ± 0.002	0.012 [0.005; 0.019]
CHUKH	11	*n* = 2 from Kharzinova et al. [4] *n* = 9 the current study	1.587 ± 0.006	0.175 ± 0.002	0.179 ± 0.002	0.016 [0.009; 0.023]
EVN	13	*n* = 7 from Kharzinova et al. [4] *n* = 6 the current study	1.654 ± 0.005	0.184 ± 0.002	0.19 ± 0.002	0.027 [0.02; 0.034]
EVK_YAK	12	Kharzinova et al. [4]	1.666 ± 0.005	0.189 ± 0.002	0.192 ± 0.002	0.012 [0.005; 0.019]
EVK_KRA	19	The current study	1.626 ± 0.005	0.181 ± 0.002	0.185 ± 0.002	0.014 [0.008; 0.02]
NEN_YMLN	13	The current study	1.61 ± 0.005	0.179 ± 0.002	0.183 ± 0.002	0.019 [0.012; 0.026]
NEN_NAO	64	*n* = 33 from Kharzinova et al. [4] *n* = 31 the current study	1.621 ± 0.005	0.181 ± 0.002	0.184 ± 0.002	0.014 [0.01; 0.018]
NEN_KOMI	13	The current study	1.615 ± 0.005	0.18 ± 0.002	0.184 ± 0.002	0.015 [0.008; 0.022]
NEN_ARKH	10	The current study	1.593 ± 0.006	0.178 ± 0.002	0.179 ± 0.002	0.014 [0.006; 0.022]
NEN_MUR	20	Kharzinova et al. [4]	1.645 ± 0.005	0.183 ± 0.002	0.188 ± 0.002	0.017 [0.012; 0.022]
WLD_YAK	11	The current study	1.678 ± 0.005	0.184 ± 0.002	0.19 ± 0.002	−0.023 [−0.03; −0.016]
WLD_TMR	21	Kharzinova et al. [4]	1.663 ± 0.005	0.184 ± 0.002	0.187 ± 0.002	−0.011 [−0.016; −0.006]

^1^ CHU, Chukotka; CHUKH, Chukotka–Khargin; EVN, Even; EVK_YAK, Evenk of the Yakutia region; EVK_KRA, Evenk of the Krasnoyarsk region; NEN_YMLN, Nenets of the Yamalo-Nenets Autonomous district; NEN_NAO, Nenets of the Nenets Autonomous district; NEN_KOMI, Nenets of the Komi Republic; NEN_ARKH, Nenets of the Arkhangelsk region; NEN_MUR, Nenets of the Murmansk region; WLD_YAK, wild reindeer of Northern Yakutia; WLD_TMR, wild reindeer of Western Taymyr. ^2^ *n*, sample size. ^3^ Source of sampling. ^4^ A_R_, allelic richness. ^5^ H_O_, observed heterozygosity. ^6^_U_H_E_, unbiased expected heterozygosity. ^7^ F_IS_, inbreeding coefficient based on the difference between _U_H_E_ and H_O_ with a 95% confidence interval (CI; in square brackets).

**Table 2:** Genetic differentiation of domestic reindeer breeds measured by *F*_ST_ values.

^1^ Breeds/ Populations	CHU	CHUKH	EVN	EVK_ YAK	EVK_ KRA	NEN_ YMLN	NEN_ NAO	NEN_ KOMI	NEN_ ARKH	NEN_ MUR	YAK	TM
**CHU**	0.000											
**CHUKH**	0.01	0.000										
**EVN**	0.051	0.045	0.000									
**EVK_** **YAK**	0.053	0.046	0.017	0.000								
**EVK_** **KRA**	0.075	0.07	0.04	0.029	0.000							
**NEN_** **YMLN**	0.086	0.08	0.052	0.04	0.016	0.000						
**NEN_** **NAO**	0.085	0.08	0.053	0.042	0.018	0.004	0.000					
**NEN_** **KOMI**	0.086	0.08	0.051	0.041	0.017	0.003	0.005	0.000				
**NEN_** **ARKH**	0.096	0.09	0.061	0.049	0.027	0.013	0.011	0.012	0.000			
**NEN_** **MUR**	0.078	0.073	0.047	0.038	0.023	0.014	0.014	0.013	0.022	0.000		
**YAK**	0.075	0.07	0.054	0.049	0.056	0.061	0.063	0.06	0.069	0.042	0.000	
**TMR**	0.074	0.068	0.054	0.049	0.053	0.058	0.058	0.056	0.066	0.038	0.005	0.000

^1^ CHU, Chukotka; CHUKH, Chukotka–Khargin; EVN, Even; EVK_YAK, Evenk of the Yakutia region; EVK_KRA, Evenk of the Krasnoyarsk region; NEN_YMLN, Nenets of the Yamalo-Nenets Autonomous district; NEN_NAO, Nenets of the Nenets Autonomous district; NEN_KOMI, Nenets of the Komi Republic; NEN_ARKH, Nenets of the Arkhangelsk region; NEN_MUR, Nenets of the Murmansk region; WLD_YAK, wild reindeer of Northern Yakutia; WLD_TMR, wild reindeer of Western Taymyr.

## Supplementary Materials

The following are available online at https://www.mdpi.com/2076-2615/10/8/1309/s1, Figure S1. The photo of the male of Nenets breed provided by Romanenko T.M., Figure S2. The photo of the male of Evenk breed provided by Goncharov V.V., Figure S3. The photo of the herd of the Even breed provided by Fedorov V.I., Figure S4. The photo of the herd of the Chukotka breed provided by Bryzgalov G.Ya., Figure S5. Calculated Cross Validation errors for each K from 2 to 10 for studied reindeer breeds.
