# Supplementary figures and images for: Insight into the Current Genetic Diversity and Population Structure of Domestic Reindeer (Rangifer tarandus) in Russia
Source: Animals (Basel). 2020 Jul 30;10(8):1309. doi: 10.3390/ani10081309 (PMC7459450; doi:10.3390/ani10081309)

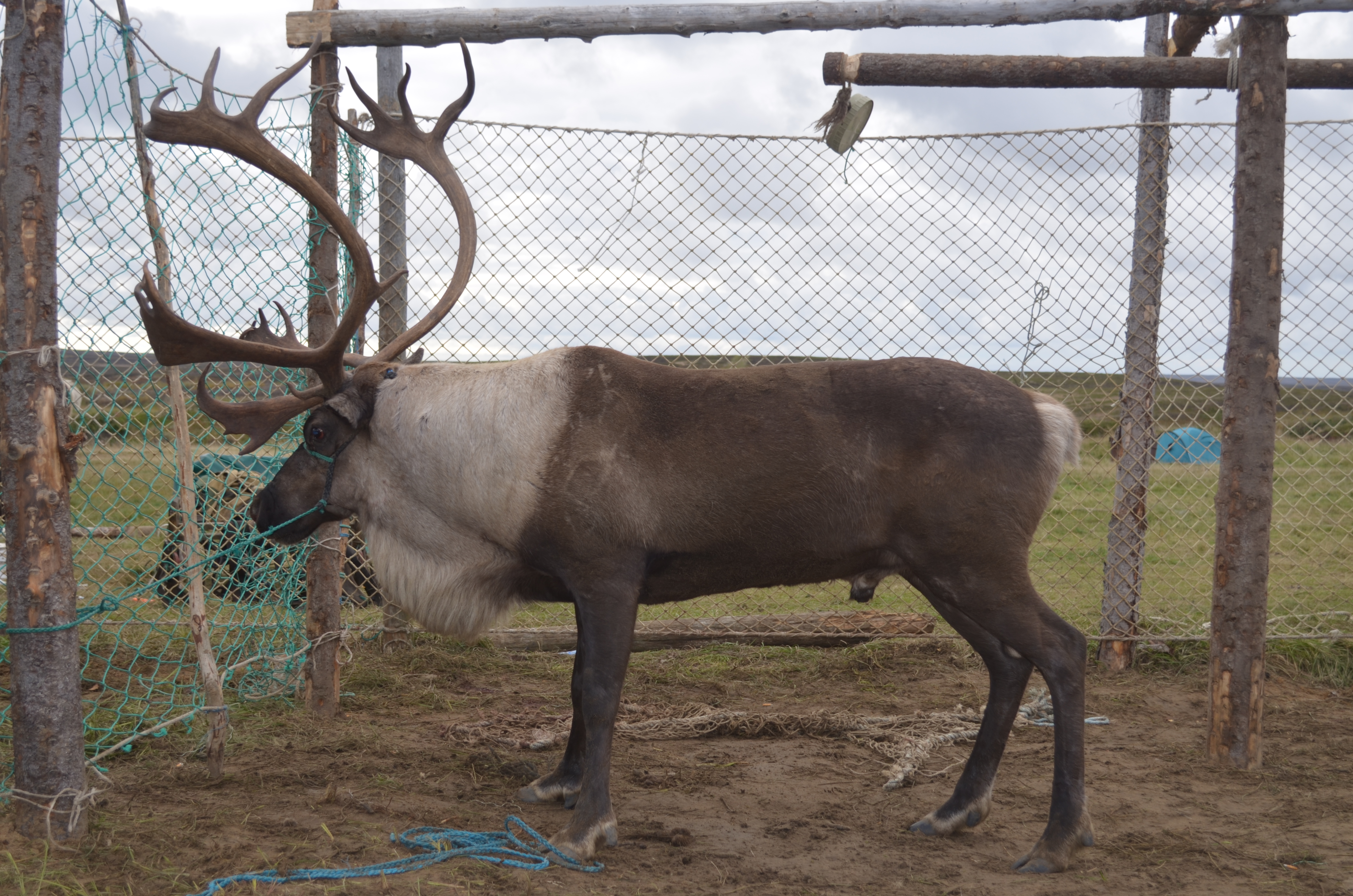

Supplement: Supplementary file 1 [file animals-10-01309-s001.zip › Supplementary files_Figures and bed_bim_fam_files/Figure S1.JPG]

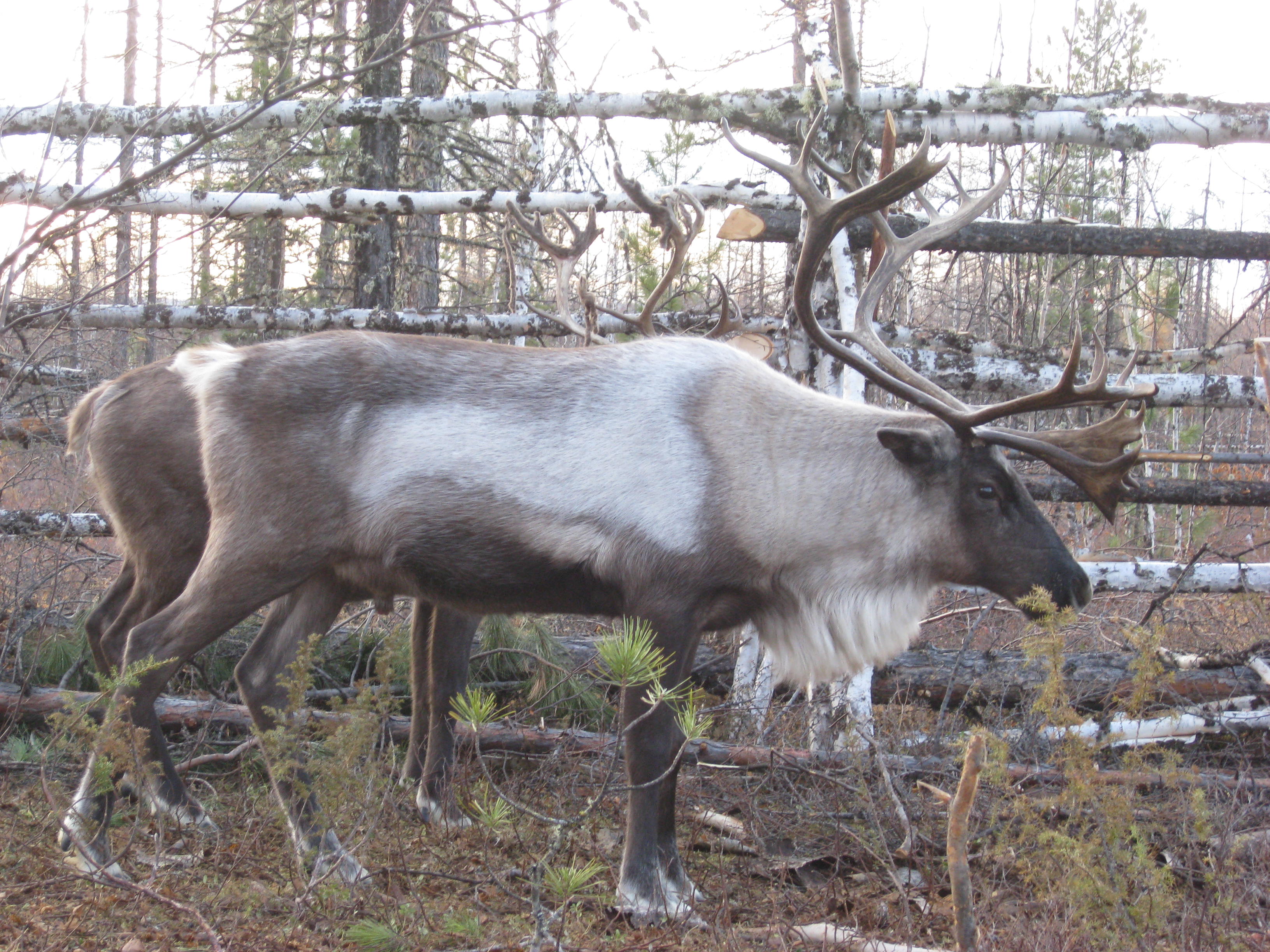

Supplement: Supplementary file 1 [file animals-10-01309-s001.zip › Supplementary files_Figures and bed_bim_fam_files/Figure S2.JPG]

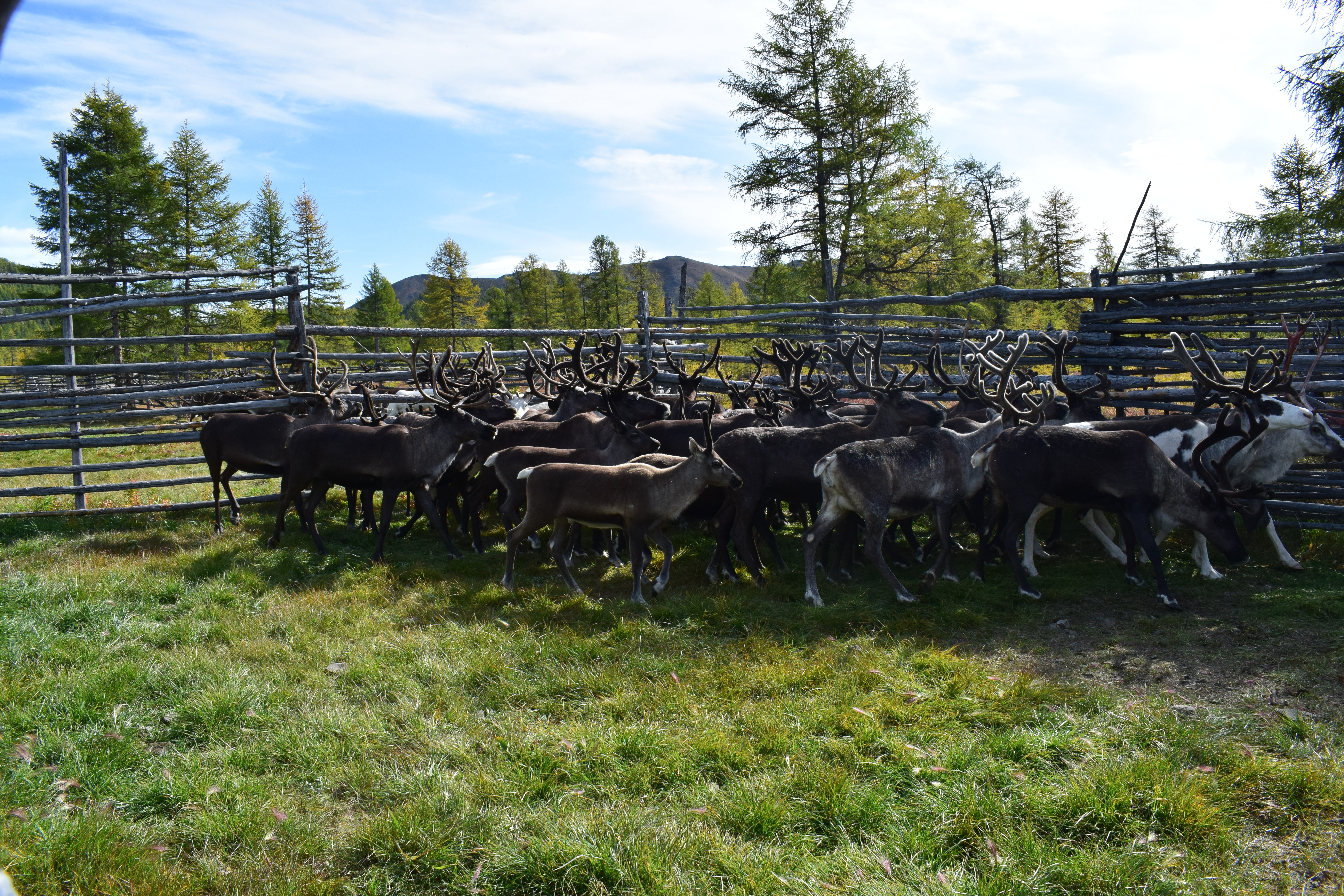

Supplement: Supplementary file 1 [file animals-10-01309-s001.zip › Supplementary files_Figures and bed_bim_fam_files/Figure S3.JPG]

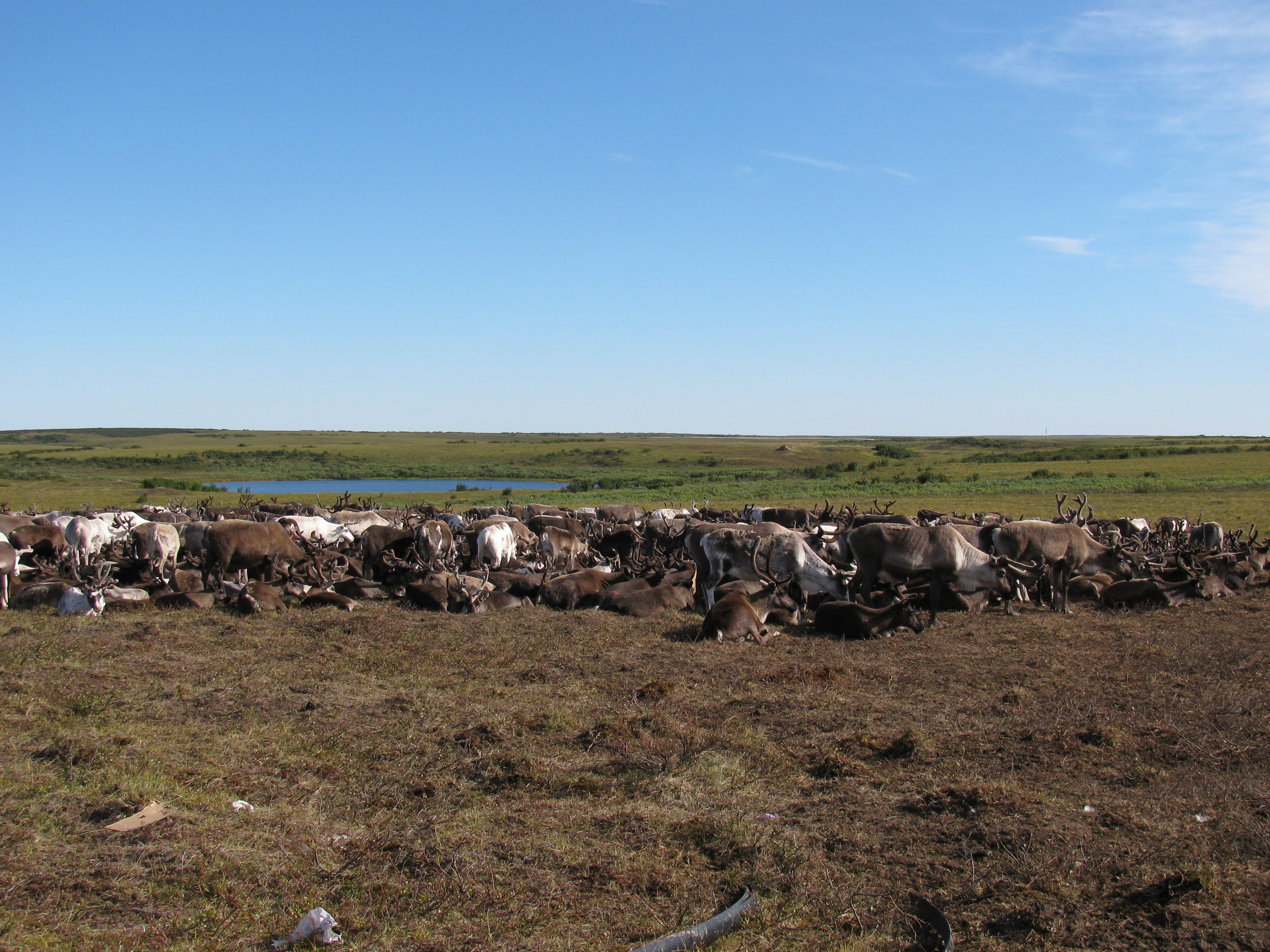

Supplement: Supplementary file 1 [file animals-10-01309-s001.zip › Supplementary files_Figures and bed_bim_fam_files/Figure S4.JPG]

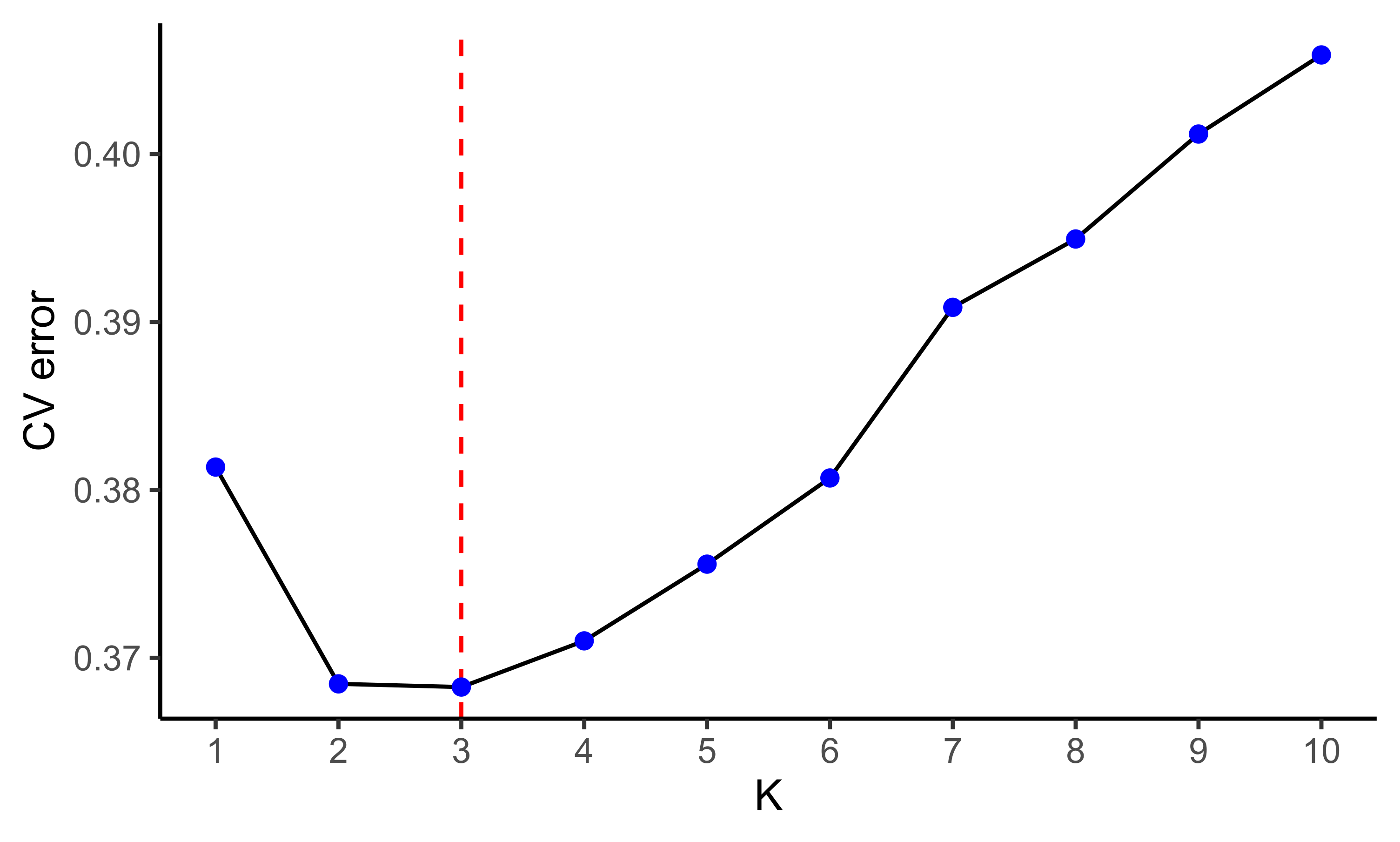

Supplement: Supplementary file 1 [file animals-10-01309-s001.zip › Supplementary files_Figures and bed_bim_fam_files/Figure S5.png]
